# Ingestion of a THC-Rich Cannabis Oil in People with Fibromyalgia: A Randomized, Double-Blind, Placebo-Controlled Clinical Trial

**DOI:** 10.1093/pm/pnaa303

**Published:** 2020-10-28

**Authors:** Carolina Chaves, Paulo Cesar T Bittencourt, Andreia Pelegrini

**Affiliations:** Public Health School, Municipal Health Secretary, Florianopolis, Brazil; Department of Internal Medicine, Federal University of Santa Catarina, Florianopolis, Brazil; Department of Physical Education, Santa Catarina State University, Florianopolis, Brazil

**Keywords:** Cannabis, Chronic Pain, Fibromyalgia, Marijuana, Pain, Tetrahydrocannabinol

## Abstract

**Objective:**

To determine the benefit of a tetrahydrocannabinol (THC)-rich cannabis oil on symptoms and quality of life of fibromyalgia patients.

**Methods:**

A double-blind, randomized, placebo-controlled clinical trial was conducted for eight weeks to determine the benefit of a THC-rich cannabis oil (24.44 mg/mL of THC and 0.51 mg/mL of cannabidiol [CBD]) on symptoms and quality of life of 17 women with fibromyalgia, residents of a neighborhood with a low socioeconomic profile and a high incidence of violence in the city of Florianopolis, Brazil. The initial dose was one drop (∼1.22 mg of THC and 0.02 mg of CBD) a day with subsequent increases according to symptoms. The Fibromyalgia Impact Questionnaire (FIQ) was applied at pre- and postintervention moments and in five visits over eight weeks.

**Results:**

There were no significant differences on baseline FIQ score between groups. However, after the intervention, the cannabis group presented a significant decrease in FIQ score in comparison with the placebo group (*P* = 0.005) and in comparison with cannabis group baseline score. (*P* < 0.001). Analyzing isolated items on the FIQ, the cannabis group presented significant improvement on the “feel good,” “pain,” “do work,” and “fatigue” scores. The placebo group presented significant improvement on the “depression” score after intervention. There were no intolerable adverse effects.

**Conclusions:**

Phytocannabinoids can be a low-cost and well-tolerated therapy to reduce symptoms and increase the quality of life of patients with fibromyalgia. Future studies are still needed to assess long-term benefits, and studies with different varieties of cannabinoids associated with a washout period must be done to enhance our knowledge of cannabis action in this health condition.

## Introduction

Fibromyalgia (FM) is one of the most common chronic pain syndromes, characterized by musculoskeletal pain, extreme fatigue, and sleep and/or mood disorders. It may have a great physical and psychological impact on patients’ lives, preventing work and daily activities. The pathophysiology is mostly unknown, and FM’s etiology involves environmental and genetic factors [1]. The disease affects more women than men, and the Brazilian Rheumatology Association calculates its prevalence in the Brazilian population at about 3%, mostly in women between 30 and 55 years old [2].

Treatment of the condition is based on symptom relief; nevertheless, modest results are obtained with current medications; however, the adverse effects of drugs often hinder patient adherence. In general, poor well-being and quality of life are common [3].

The cannabis plant has been used in pain treatment for centuries; in the last decades, however, its use has been withdrawn from traditional medicine due to legal prohibitions [4]. Scientific studies on the plant’s therapeutic effects have been produced over the last 50 years, and in 2017 the National Academy of Science, Engineering and Medicine concluded, after article reviews, that cannabis use for pain treatment is supported by well-controlled clinical trials, with substantial evidence of its effects in the treatment of chronic pain in adults [5]. Presently, many countries have been reformulating their laws recognizing the medicinal character of this plant.

Cannabis flowers contain over 100 types of phytocannabinoids, chemical compounds that interact with the cannabinoid receptors present in our bodies. The most prevalent and better known are tetrahydrocannabinol (THC) and cannabidiol (CBD). THC is found in greater quantity in drug-type cannabis chemovars and is responsible for its popular psychoactive effects: euphoria, lightheadedness, and coordination and recent memory impairment. Beneficial effects are seen on pain control, nausea, anxiety, insomnia, anorexia, and spasticity. CBD presents anti-epileptic, analgesic, anxiolytic, and sedative effects with fewer psychoactive effects. Both phytocannabinoids have anti-inflammatory effects, which seems to be greater when both THC and CBD act together [6].

There are two well-characterized endocannabinoid receptors on animals: CB1 and CB2; CB1 is mostly found in the central nervous system, while CB2 is found in many tissues and organs, especially those with immune-related activities [3]. There is also growing evidence that support the existence of additional receptors for cannabinoids (non-CB1/CB2). This set of receptors, along with endocannabinoid substances (2-arachidonoylglycerol and anandamide) and the associated biochemical apparatus, composes the endocannabinoid system [4]. This system has multiple functions that keep balance in our bodies, including pain and stress modulation, suggesting that its manipulation may be a potential therapeutic focus in FM care.

Considering the far-reaching damage caused by FM and the effect it can have on individuals, their families, communities, and the public health system, it seems necessary to study alternative, low-cost, and well-tolerated therapies that help patients to regain their well-being and quality of life. The present study aims to evaluate the impact that cannabis oil—a THC-rich whole plant extract—can have on symptoms and quality of life of individuals afflicted by FM.

## Methods

### Study Design

A randomized, double-blind, placebo-controlled clinical trial performed with patients of a community Health Center in Florianopolis, Brazil. Data were collected from September to November of 2019.

### Participants

Users of Monte Cristo Health Center with fibromyalgia or chronic spread pain were recruited by family health teams to participate. Twenty people attended a pre-evaluation meeting with the researcher and signed a written informed consent form that enabled access to their medical records. Diagnosis of fibromyalgia was confirmed at this meeting using American College of Rheumatology (ACR) 2010 criteria [7, 8]. Inclusion criteria were FM diagnosis (ACR 2010 criteria), age over 18 years, presence of moderate to severe symptoms (presenting functional limitation in everyday activities) despite therapies in use, at least one medical or nursing consultation at the Health Center in the last year. Exclusion criteria were decompensated organic comorbidities and/or risk of psychiatric conditions (schizophrenia, psychosis, severe personality disorder, current suicidal ideation), another well-defined cause of chronic pain, current pregnancy/lactation, moderate or severe cognitive impairment, and history of cannabinoid sensitivity.

### Randomization and Masking

Participants were randomly assigned through a computer randomization program to two groups, cannabis or placebo, with the help of an external collaborator. This same person was not involved in the rest of the trial and kept allocation information in sealed opaque envelopes until the end of the intervention. The cannabis group received a 30-mL green glass dropper bottle containing cannabis oil (olive oil extraction) of the White Widow [9] variety, at a 24.44-mg/mL concentration of THC and 0.51 mg/mL of CBD—at a proportion of ∼48/1 THC/CBD, among small quantities of other cannabinoids such as cannabigerol, tetrahydrocannabivarin, cannabinol, and cannabicromen. The material was not analyzed for terpenoid profile, although the chosen variety of cannabis is recognized for its terpenoids: myrcene, caryophyllene, and pinene [9]. The product—not standardized—was provided in partnership with the Brazilian Association of Medical Cannabis Patients (AMA+ME). The placebo group received an identical bottle with olive oil inside. Edible brown dye was used to soften differences between liquids. Participants were informed about the possibility of being placed into the placebo group. They were evaluated during the study with prescheduled individual appointments to reduce information exchange at the Monte Cristo Health Center. The main researcher made all the evaluations while blinded. Data analysis was conducted after the end of the intervention.

### Procedures and Outcome

The initial dose in both groups was one drop (∼1.2 mg of THC and 0.02 mg of CBD) a day sublingually. Participants in both groups were seen at baseline and every 10 days for eight weeks, and dose increases respected the maximum of one drop for each evaluation moment. At each visit, patients filled out the Fibromyalgia Impact Questionnaire (FIQ), a validated self-administered test that evaluates physical function, work status, well-being, and associated physical and mental symptoms in FM patients [10]. The FIQ is composed of 10 items (“physical function,” “feel good,” “work missed,” “job ability,” “pain,” “fatigue,” “morning tiredness,” “stiffness,” “anxiety,” and “depression”), each with a maximum possible score of 10. Total scores range from 0 to 100, and higher scores mean greater impact on a patient’s quality of life [11]. Clinical and adverse effects were also assessed during each visit to determine change or maintenance of therapy dosage.

### Statistical Analysis

Data are presented as mean and SD. Analysis of repeated measures was elected to compare mean values in the questionnaire on five visits during the intervention, excluding pre- and postintervention measures. The Mauchly test was used to verify the sphericity of the data (*P* = 0.593 for the cannabis group and *P* = 0.439 for the placebo group). Comparison of mean values between groups pre-intervention was verified using the Mann-Whitney test. The Wilcoxon rank test was chosen to verify differences in mean values pre- and postintervention for each group separately. Data were analyzed using IBM SPSS, version 20.0, with *P* values <0.05 considered significant.

## Results

**Figure 1.**
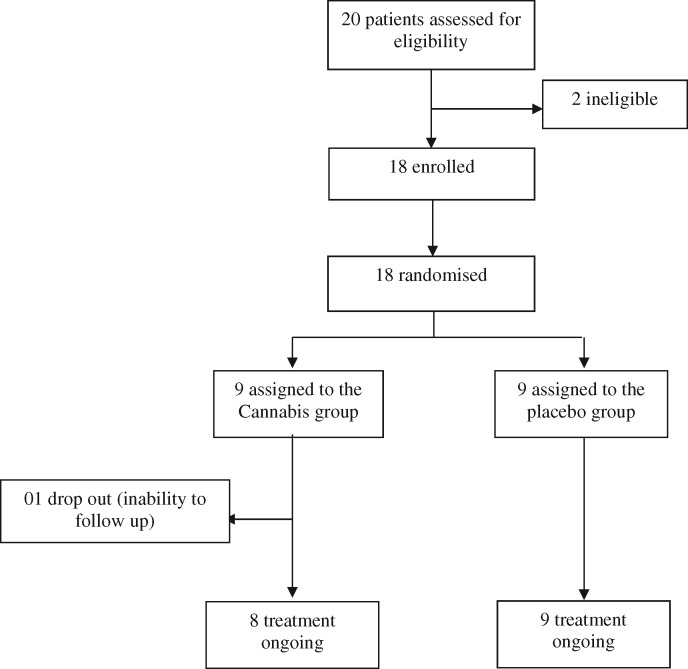
Trial profile.

Twenty people attended the first meeting after family health team recruitment on September 27, 2019. One person did not meet criteria for fibromyalgia diagnosis, and another was excluded due to psychiatric risk. Eighteen people were found to be eligible for participation. Two days after the pre-intervention meeting, one participant from the cannabis group dropped out, reporting inability to attend most intervention evaluations. Seventeen participants finished the study (eight in the cannabis group and nine in the placebo group), which occurred between September 27 and November 25 of 2019 (Figure 1).

The mean age was 51.9 years, and all participants were women (100%) residents of a neighborhood with a low socioeconomic profile and a high incidence of violence in the city of Florianopolis, Brazil. Participants in the cannabis group had previously used antidepressants (62.5%), opioids (25%), and benzodiazepines (12.5%). In the placebo group, the rates of the same class of medications use were 67%, 33%, and 11%, respectively. Patients self-medicated with mild analgesics and anti-inflammatory pills whenever necessary in both groups.

Participants started intervention with one drop a day (∼1.2 mg of THC and 0.02 mg of CBD), and doses could be increased throughout evaluations, which occurred every 10 days. The mean dose at postintervention evaluation was 3.6 drops of cannabis oil (∼4.4 mg of THC and 0.08 mg of CBD) in the cannabis group and 4.3 drops of olive oil in the placebo group. The effects reported by the cannabis group were somnolence (87.5%), dizziness (25%), mouth dryness (25%), improved mood (25%), and improved libido (12.5%). One participant (11%) in the placebo group related somnolence during the 12 hours after product intake. Change in sleep pattern was considered a positive effect in the cannabis group, given that most participants suffered from insomnia or nonrestorative sleep. There was no follow-up loss by unpleasant adverse effects.

Pre-intervention, there were no significant differences between groups on FIQ mean scores. However, after eight weeks, the cannabis group presented a statistically lower total score on the FIQ compared with the placebo group (*P* = 0.005). Analyzing isolated items of the FIQ, it was observed during the pre-intervention evaluation that there was a difference between groups only on the “physical impairment” item, with greater values in the cannabis group (greater impairment). After intervention, statistical differences were observed in mean values on the “feel good,” “do work,” and “pain” items. These results indicated lower values for the cannabis group in comparison with the placebo group (Table 1).

**Table 1.** Comparison of mean scores on FIQ between groups

Study Variable	Pre-intervention		*P* Value	Postintervention		*P* Value*
	Cannabis	Placebo		Cannabis	Placebo	
	$\bar{\text{x}}\;\left(\text{sd}\right)$	$\bar{\text{x}}\;\left(\text{sd}\right)$		$\bar{\text{x}}\;\left(\text{sd}\right)$	$\bar{\text{x}}\;\left(\text{sd}\right)$	
FIQ (0–100)	75.50 (12.93)	70.22 (11.18)	0.381	30.50 (16.18)	61.22 (17.30)	0.005
Physical function (0–10)	6.37 (1.88)	4.03 (2.08)	0.021	5.83 (2.02)	4.07 (2.25)	0.139
Feel good (0–10)	9.47 (1.06)	9.68 (0.95)	0.673	1.73 (0.64)	7.50 (2.93)	0.002
Work missed (0–10)	5.10 (3.86)	7.14 (4.95)	0.517	2.38 (1.65)	6.57 (3.29)	0.071
Job ability (0–10)	7.13 (2.90)	7.89 (2.15)	0.606	4.29 (1.70)	7.89 (1.36)	0.001
Pain (0–10)	8.25 (1.98)	8.67 (2.96)	0.481	3.75 (2.49)	7.67 (1.84)	0.006
Fatigue (0–10)	8.00 (2.07)	7.33 (3.39)	0.963	4.00 (2.08)	6.11 (3.37)	0.174
Morning tiredness (0–10)	7.88 (1.42)	8.33 (2.06)	0.815	4.50 (1.91)	7.67 (3.16)	0.106
Stiffness (0–10)	7.75 (2.05)	6.11 (2.84)	0.236	3.33 (3.21)	5.00 (3.91)	0.482
Anxiety (0–10)	8.38 (1.69)	8.00 (2.00)	0.743	7.00 (2.92)	7.00 (2.87)	0.898
Depression (0–10)	7.50 (2.45)	7.78 (2.49)	0.815	5.80 (3.11)	4.67 (3.84)	0.699

FIQ = Fibromyalgia Impact Questionnaire; $\bar{\text{x}}\text{ average value}$; sd = standard deviation.

* Mann-Whitney test (nonparametric data). There were significant reductions in total FIQ score and on the “feel good,” “job ability,” and “pain” items in the cannabis group compared with the placebo group. The cannabis group initially presented a significantly greater score on the “physical impairment” item.

Comparing pre- and postintervention FIQ mean scores in each group, the cannabis group presented a statistically significant reduction, going from 75.5 to 30.5 points (*P* < 0.001). At the same time, the placebo group maintained its score (*P* = 0.07). Furthermore, in an isolated analysis of FIQ items, the cannabis group presented a reduction in mean values on the “feel good,” “pain,” and “fatigue” items. The placebo group presented a reduction in mean values on the “depression” item (Table 2).

**Table 2.** Comparison of FIQ mean scores pre- and postintervention in both groups

Study Variable	Cannabis		*P* Value	Placebo		*P* Value
	Pre	Post		Pre	Post	
	$\bar{\text{x}}\;\left(\text{sd}\right)$	$\bar{\text{x}}\;\left(\text{sd}\right)$		$\bar{\text{x}}\;\left(\text{sd}\right)$	$\bar{\text{x}}\;\left(\text{sd}\right)$	
FIQ (0–100)	75.50 (12, 93)	30.50 (16, 18)	<0.001	70.22 (11, 18)	61.22 (17, 30)	0.070
Physical function (0–10)	6.37 (1.88)	5.83 (2.02)	0.109	4.03 (2.08)	4.07 (2.25)	0.495
Feel good (0–10)	9.47 (1.06)	1.72 (0.64)	0.039	9.68 (0.95)	7.50 (2.93)	0.104
Work missed (0–10)	5.10 (3.86)	2.38 (1.65)	0.317	7.14 (4.95)	6.57 (3.29)	0.317
Job ability (0–10)	7.13 (2.90)	4.29 (1.70)	0.093	7.89 (2.15)	7.89 (1.36)	0.831
Pain (0–10)	8.25 (1.98)	3.72 (2.49)	0.011	8.67 (2.96)	7.67 (1.87)	0.235
Fatigue (0–10)	8.00 (2.07)	4.00 (2.08)	0.027	7.33 (3.39)	6.11 (3.37)	0.112
Morning tiredness (0–10)	7.88 (2.42)	4.50 (1.91)	0.257	8.33(2.06)	7.67(3.16)	0.465
Stiffness (0–10)	7.75 (2.05)	3.33 (3.21)	0.285	6.11 (2.84)	5.00 (3.91)	0.512
Anxiety (0–10)	8.38 (1.69)	7.00 (2.91)	0.135	8.00 (2.00)	7.00 (2.87)	0.397
Depression (0–10)	7.50 (2.45)	5.80 (3.11)	0.465	7.78 (2.49)	4.67 (3.84)	0.027

Wilcoxon rank test. There were significant reductions in total FIQ score and on the “feel good,” “pain,” and “fatigue” items in the cannabis group after intervention. The placebo group presented a significant reduction on the “depression” item after intervention.

FIQ = Fibromyalgia Impact Questionnaire; $\bar{\text{x}}\text{ average value}$; sd = standard deviation.

During the intervention period, three patients in the cannabis group (37.5%) reported better disposition for functional activities such as cooking and housekeeping, and another patient (12.5%) reported feeling more comfortable with accomplishing her professional activity (seamstress).

It was observed that the mean score on the FIQ in both groups was maintained during most of the evaluation visits throughout intervention, with no significant variation in subsequent visits, except between visit 1 and visit 4 in the cannabis group (*P* = 0.032), revealing that the perception of quality of life at time 4 was statistically better compared with time 1. In the placebo group, no difference was observed between moments (*P* > 0.05) (Table 3).

**Table 3.** Analysis of repeated measures of FIQ score in both groups at evaluation visits throughout intervention

Visits	Cannabis	Placebo
	$\bar{\text{x}}\;\left(\text{sd}\right)$	$\bar{\text{x}}\;\left(\text{sd}\right)$
V1	57.50 (12.91)*	58.56 (19.78)
V2	48.88 (19.21)*^,†^	51.67 (17.59)
V3	47.75 (16.75)*^,†^	52.33 (23.44)
V4	42.13 (17.18)^†^	58.11 (19.79)
V5	43.25 (24.41)*^,†^	53.44 (21.14)

Analysis of repeated measures (distinct symbols represent significant differences between the moments in the mean values)

The mean score on the FIQ in both groups was maintained during most of evaluation visits throughout intervention; there was no significant variation in subsequent visits, except between visit 1 and visit 4 in the cannabis group (*P* = 0.032). In the placebo group, no difference was observed between moments.

FIQ = Fibromyalgia Impact Questionnaire; V1 = first visit; V2 = second visit; V3 = third visit; V4 = fourth visit; V5 = fifth visit; $\bar{\text{x}}\text{ average value}$; sd = standard deviation

## Discussion

In the present study, we investigated the impact of a THC-rich cannabis oil on quality of life and symptoms of people with fibromyalgia, residents of the same neighborhood in the city of Florianopolis, Brazil. After eight weeks of trial, a statistically significant reduction in FIQ score was observed in the cannabis group (*P* < 0.001). The cannabis group also presented a statistically significant reduction in FIQ score compared with the placebo group (*P* = 0.005).

The baseline score on the FIQ in our study is considered high in comparison with other studies, indicating greater impact on the quality of life of these women [12–14]. We believe that the low socioeconomic level and high incidence of violence in their community may have potentialized this phenomenon. In this context, our findings suggest that phytocannabinoids are effective in treating people with FM, including those with severe symptoms.

The reduction in FIQ score in the cannabis group is similar to findings from other studies with allopathic therapy [12–17]. In the present study, however, we observed an extremely significant reduction on FIQ (*P* < 0.001) with cannabis oil, while allopathic therapy studies presented more modest reductions on FIQ and/or greater incidence of intolerable adverse effects, leading to considerable rates of participant dropout.

The oil used in this research was an integral extract of cannabis with a higher content of THC (tetrahydrocannabinol) than CBD (cannabidiol). This choice was based on the fact that THC is one of the most studied phytocannabinoids, with therapeutic potential already demonstrated in chronic and oncologic pain treatment, intractable pruritus, nausea, anorexia, and mood swings related to chemotherapy. Additionally, German researchers evaluated the analgesic effects of THC administered orally in nine patients with FM over a period of three months, using daily doses of 2.5–15 mg of THC without other analgesic medications. All participants who finished the study reported pain reduction, both in daily symptom records and after experimentally induced pain sessions [18]. A synthetic cannabinoid that mimics THC (Nabilone) also demonstrated pain reduction and improvement in quality of life in people with FM in a randomized, double-blind, placebo-controlled trial with 40 participants [17]. A recent experimental randomized study with chronic pain patients demonstrated analgesic efficacy of inhaled pharmaceutical-grade cannabis varieties containing THC in the evoked pressure pain model, when compared to placebo [19].

The mean dose used in this study was 3.6 drops a day (∼4.4 mg of THC and 0.08 mg of CBD) in the cannabis group, and adverse events were attenuated over time. We suggest starting treatment with low doses of THC (∼1 mg), with subsequent increases according to clinical response, in order to achieve good results with the lowest dose required. Due to the short intervention time (eight weeks), participants were not instructed to cease or reduce other medications used in FM treatment; there was, though, spontaneous reduction of antidepressant (three patients) and benzodiazepine medication (one patient) in the cannabis group during intervention, a finding already associated with cannabis use in the literature [1].

One main complaint of patients with FM is chronic widespread pain, and some of them suffer from concomitant symptoms, such as fatigue, morning stiffness, mood, and sleep disturbance [7]. During the intervention, the impact of the intervention on quality of life in the cannabis group participants was evident, resulting in reports of well-being and more energy for activities of daily living. Pain attacks were also reduced, albeit subjectively, in frequency and intensity. These results were expected due the therapeutic effects of cannabis already demonstrated in other trials [3, 5, 18].

Our analysis of FIQ isolated items confirmed clinical evidence. Statistically significant improvements on the “feel good,” “do work,” and “pain” items in the cannabis group were found upon analysis between groups, and an additional improvement on the “fatigue” item was found in this same group compared with its baseline value. Findings on pain reduction are already established [1, 3, 5, 17, 18], although the main cause of this benefit remains unclear, as cannabinoids act at many sites along pain transmission pathways [17].

The significant improvement on the “feel good” item in the cannabis group was extremely motivating to us, considering that this item had the greatest score of the FIQ items in both groups pre-intervention, and after intervention, it became the lowest score on FIQ items in the cannabis group but maintained its value in the placebo group. Also, we can extrapolate the importance of this subjective feeling of well-being according to the World Health Organization definition of health: a state of complete physical, mental, and social well-being and not merely the absence of disease or infirmity [20]. Findings of improvement on the “fatigue” and “do work” scores also support our belief in the potential of cannabis to enhance quality of life in FM patients.

The reduction in “depression” score in the placebo group after the intervention made us question the effectiveness of this scale to evaluate mood impairment (anxiety and depression), as these single items of the questionnaire are not validated scales for respective symptoms [17]. We believe that significant and rare insight into one’s personal psychological state is necessary to assess these topics, so we also suggest that validated scales are probably more suitable for the assessment of psychological symptoms [17].

Finally, the significant difference in FIQ scores between visit 1 and visit 4 in the cannabis group led us to question the time needed for cannabis therapy to demonstrate significant effects, considering that significant differences were maintained at the final evaluation (postintervention). Although visit 5 did not demonstrate a significant reduction in comparison with visit 1, we observed that the standard deviation at this fifth visit was greater than others. The fluctuating character of FM symptoms, with exacerbation periods, in addition to a greater susceptibility in terms of environmental disturbances (remembering that the participants lived in a community with a high incidence of violence), may contribute to this isolated fact.

We consider important to understand FM as a pain syndrome with multiple etiologies (central sensitization, altered stress response, pro-inflammatory state, abnormal activity of neurotransmitters, small-fiber peripheral neuropathy, genetic predisposition), where cannabis can act in different ways. Cannabinoids reduce pain and other pathophysiological and physiological processes through varied mechanisms involving its receptors in the organisms. The endocannabinoid system consists of cannabinoid receptors (mostly known as CB1 and CB2), the endocannabinoid substances, and their biosynthetic and catabolic enzymes. It is active in the central nervous system and in the peripheral nervous system, modulating pain on the spinal, supraspinal, and peripheral levels; endocannabinoids substances—anandamide and 2-arachidonoylglycerol—are produced on demand in these systems to contain hyperalgesia, allodynia, and inflammatory states. There is also good evidence that cannabinoid receptors play a role in the modulation of neurotransmitters such as serotonin, dopamine, and others [3, 18, 21, 22].

Some studies suggest that pathological conditions in pain modulation such as fibromyalgia, migraine, and irritable bowel syndrome, among others, may be, at least in part, related to the deregulation of the endocannabinoid system. In this context, manipulation of the endocannabinoid system, which is associated with the immunomodulatory effect of cannabinoids, strengthens the role of these compounds as promising therapeutic agents [21, 23].

Despite the significant findings of this medical residency’s final work, there are limitations to acknowledge: small sample size, short intervention period, participants maintained other FM treatments, cannabis oil and the placebo product did not have the same color (brown vs light brown, respectively) and were not masked for taste differences, and participants were individually evaluated by the main researcher during all intervention periods—although both were blinded.

## Conclusions

To our knowledge, this is the first randomized controlled trial to demonstrate the benefit of cannabis oil—a THC-rich whole plant extract—on symptoms and on quality of life of people with fibromyalgia. We conclude that phytocannabinoids can be a low-cost and well-tolerated therapy for symptom relief and quality of life improvement in these patients, and we suggest that this therapy could be included as an herbal medicine option for the treatment of this condition in the Brazilian public health system.

Larger and longer studies, accessing integral extracts of cannabis with varied concentrations between phytocannabinoids and including a washout period, must be done to enhance our knowledge about cannabis action in fibromyalgia.

## Notes

Funding sources: There was no funding source for this study.
